# The Influence of Situational Regulation on the Information Processing of Promotional and Preventive Self-Regulatory Individuals: Evidence From Eye Movements

**DOI:** 10.3389/fpsyg.2020.531147

**Published:** 2020-11-17

**Authors:** Jianping Xiong, Xiaokang Jin, Weili Li

**Affiliations:** Department of Psychology, Faculty of Education, Henan Normal University, Xinxiang, China

**Keywords:** chronic regulatory focus, situational regulatory focus, information processing, alternative-based information, attribute-based information, eye movements

## Abstract

Regulatory focus theory uses two different motivation focus systems—promotional and preventive—to describe how individuals approach positive goals and avoid negative goals. Moreover, the regulatory focus can manifest as chronic personality characteristics and can be situationally induced by tasks or the environment. The current study employed eye-tracking methodology to investigate how individuals who differ in their chronic regulatory focus (promotional vs. preventive) process information (Experiment 1) and whether an induced experimental situation could modulate features of their information processing (Experiment 2). Both experiments used a 3 × 3 grid information-processing task, containing eight information cells and a fixation cell; half the information cells were characterized by attribute-based information, and the other half by alternative-based information. We asked the subjects to view the grid based on their personal preferences and choose one of the virtual products presented in this grid to “purchase” by the end of each trial. Results of Experiment 1 show that promotional individuals do not exhibit a clear preference between the two types of information, whereas preventive individuals tend to fixate longer on the alternative-based information. In Experiment 2, we induced the situational regulatory focus via experimental tasks before the information-processing task. The results demonstrate that the behavioral motivation is significantly enhanced, thereby increasing the depth of the preferred mode of information processing, when the chronic regulatory focus matches the situational focus. In contrast, individuals process information more thoroughly, using both processing modes, in the non-fit condition, i.e., when the focuses do not match.

## Introduction

The hedonic principle proposes that people characteristically approach pleasure and avoid pain. Furthermore, regulatory focus theory explains how individuals succeed in these goals. In this theory, Higgins (1997) distinguishes two motivational systems that regulate individual goal-directed behaviors: promotional and preventive. People incline toward goal means that have higher regulatory fit; specifically, individuals with promotional focus (“promoters”) incline more toward eagerness means, whereas preventive individuals (“preventers”) prefer vigilance means because individuals have stronger motivation under fitting circumstances. Moreover, the regulatory focus may be emphasized as a chronic orientation developed during socialization or as a situational inclination induced by a given situation or task. We wondered whether there was a fit or compatibility between chronic and situational regulatory focus and how the fit of these two regulatory focuses states affect the individual’s information processing in decision making. Accordingly, we first explored the information processing of individuals with differing chronic regulatory focuses when they were making decisions. Subsequently, we probed the effects on individuals’ information processing when the different chronic regulatory focuses were combined with situational regulatory focuses. Our framework was regulatory focus theory, and we obtained our data using the eye-tracking method.

### Regulatory Focus and Information-Processing Features

Regulatory focus is individuals’ application of specific means or inclinations when they pursue their goals (Manczak et al., 2014). Regulatory focus theory states that regulatory focus systems can be divided into advancement-related (promotional) focus and security-related (preventive) focus. A promotional motivation system concentrates on developmental needs and values, progress, and achievement; therefore, promoters strive to approach the ideal self, pay more attention to whether pursuit of a goal produces positive results, and tend to show eagerness in approach behaviors. In contrast, a preventive motivation system focuses on security needs and values, responsibility, and reduction of risk; therefore, preventers pursue ought-self goals, pay more attention to whether the goal pursuit could have negative outcomes, and tend to use vigilance and avoidance behaviors (Higgins, 1997, 1998; Brockner and Higgins, 2001; Florack et al., 2013).

Based on the distinctive characteristics of the two motivation focus systems, we could infer that promoters and preventers may manifest different information-processing features. Indeed, it has been found that promoters process more categories of information, compared with preventers, when they confront multiple categories of information (Chernev, 2004; Florack et al., 2010). Promoters incline more toward processing information on a global level, whereas preventers are more likely to process information on a local level. For example, Förster and Higgins (2005) found that promotion-oriented strength was positively correlated with global information processing and negatively correlated with local information processing, but prevention-oriented strength was the opposite. The study of Lee et al. (2009) indicated that promotion-focused individuals were more likely to construe information at abstract, high levels, whereas those with a prevention focus were more likely to construe information at concrete, low levels. Another study (Semin et al., 2005) found that more abstract expressions were used to describe promotion-focused friendship strategies, while more specific expressions were used for prevention-focused friendship strategies. These converging lines of evidence reveal that for the same information, promoters prefer to represent it more abstractly, while preventers are inclined to process information at a more specific level. This is because a more holistic and abstract processing pattern is conducive to obtaining more recognition opportunities and reducing omissions, which is consistent with the eager approach feature of promoters. Conversely, a more specific and local processing pattern is beneficial for acquiring more detailed information and avoiding errors as much as possible, which matches the vigilant avoidance feature of preventers.

A multi-attribute decision-making task is one of the most common decision tasks in our lives. An attribute-based processing pattern and an alternative-based processing pattern are two popular ways to search for and process information in such a task (Payne et al., 1993). The former pattern relates to the tendency of decision makers to search for a certain attribute and compare the differences between attribute values among the alternatives. On the other hand, an alternative-based processing pattern relates to the tendency to make a decision by searching for a certain alternative and comparing the values of all the attributes for this alternative.

A study by Payne et al. (1996) showed that individuals tend to use alternative-based search patterns with less selective processing when accuracy in the task is emphasized. In regulatory focus theory, accuracy is the main concern of preventers, so they are more likely to use an alternative-based pattern. Another study, by Wang and Liu (2014), also found that preventers evaluate their decisions more positively when they use an alternative-based pattern, whereas promoters evaluate their decisions more positively when they use an attribute-based search pattern. However, Sanbonmatsu and Fazio (1990) found that increased motivation to make accurate decisions prompts people to use more attribute-based information processing. Mourali and Pons (2009) also stated that attribute-based processing is more frequent when individuals are in a prevention-focus situation, whereas alternative-based processing arises more frequently in a promotion-focus situation. Although these results are divergent, all the studies support the previously mentioned conclusion that the cognitive processing of promoters occurs at a global level, and the cognitive processing of preventers occurs preventive at a more local level.

Tversky and Kahneman (1981) proposed that decision making involves two stages of cognitive processing: information coding and evaluation. Higgins (2000) also explicitly stated that self-regulatory focus and regulatory fit affect different stages of the decision-making process. However, previous studies have mainly explored the regulatory focus effect in the evaluation stage of decision making; few studies have explored the regulatory matching effect in the information-coding stage. To predict people’s judging inclinations, researchers should first understand how people comprehend and code decision-making information. Tuan Pham and Chang (2010) attempted to explore the fit effects in cognitive processing. Their study required subjects to select their favorite dishes from a menu of several different classification features. They measured the number of pages a subject opened and the total viewing time for each page to evaluate the visual information processing strategy and the subject’s involvement in the task. Viewing time, however, because of its relatively coarse nature, can only reflect an individual’s overall visual processing activities and cannot reveal the characteristics of information coding in detail during the viewing process. Previous studies (Glaholt and Reingold, 2011; Schulte-Mecklenbeck et al., 2017; Fridman et al., 2018) on the application of eye-movement technology in decision making demonstrated that a combination of multiple eye-movement measurements can accurately survey the viewer’s attention distribution and cognitive processing characteristics at different stages of decision making. Accordingly, in our first experiment, eye-movement tracking technology was employed on the individuals with different chronic regulatory focus (promotional vs. preventive) to analyze how the chronic regulatory focus affect information process before making decisions. Hypothesis 1 states that individuals with different chronic regulatory focus exhibit different information-processing features, and the chronic promoters process information at a comprehensive level, whereas chronic preventers at a local level.

### The Interaction of Chronic and Situational Regulatory Focus

Regulatory focus may manifest as a relatively stable and chronic orientation that may have developed from an individual’s early socialization or parents’ rearing methods. That is, children are more likely to develop a promotional disposition if parents pay attention to positive results and excellent behavior, whereas they may develop a disposition to prevention and avoidance if parents frequently use criticism and punishment. Regulatory focus may also manifest as a situational orientation that may be induced by a given situation or task, such as one that requires either recall of responsibilities and duties (preventive focus) or thinking of one’s hopes and beliefs (promotional focus).

Considering this, what will be the changes in the information processing of chronic promoters or preventers when they are faced with a prevention- or promotion-oriented experimental task? The regulatory fit theory (Higgins, 2005) proposes that individuals’ motivational strength will be enhanced when their pursuing goals match or fit their current regulatory focuses. When individuals complete a goal in a way that matches or fits their regulatory focus, they will feel their activities are important and valuable (Higgins et al., 2003), which should result in a stronger sense of commitment to their goal (Aaker and Lee, 2006). Such as the study of Cesario et al. (2004), the results of four experiments consistently revealed that the experience of feeling right from regulatory fit can transfer to the persuasion context, thereby influencing perceived message persuasiveness and opinion ratings. Based on this theory, we could expect further enhancement of an individual’s existing behavioral motivation when chronic regulatory focus is consistent with the situationally induced motivation orientation. Keller and Bless (2006) explored the interactive effect of chronic and situational regulation focus on cognitive test performance and found that performance was enhanced when the situationally induced motivation orientation matched the chronic self-regulatory focus of the individual tested. These results are consistent with the inference of Shah et al. (1998) and the hypothesis of regulatory fit theory, and they further demonstrate the importance of compatibility between chronic and situational self-regulatory disposition in cognitive test performance. However, a study of risk-decision tasks (Sekścińska et al., 2016) reported an inconsistent result. In this research, chronic and situationally induced motivation had no interaction in risky financial choices (the only significant interaction was observed in a gambling task in a loss-decision frame), indicating that the chronic motivational system and situationally induced motivation are independent of each other.

The aforementioned contradictory results may be due to the different nature of the experimental tasks, which may have led to different information processes. Moreover, previous related studies were all concerned of the results of motivational behavior, yet few studies have focused on the process of motivational behavior. Furthermore, the effect of the “non-fit” condition on the information processing possibly differ with that of the “fit” condition, for example, Fridman et al. (2016) found that the regulatory non-fit between the form of the physician’s advice (emphasizing gains vs. avoiding losses) and the participants’ motivational orientation (promotion vs. prevention) improved participants’ evaluation of an initially disliked option. Researchers suggested that regulatory non-fit weakened participants’ initial attitudes by making them less confident in their initial judgments and motivating them to think more thoroughly about the arguments presented, but it was unclear how the regulatory non-fit impacts the individuals’ information processing. Therefore, in our second experiment, we simultaneously manipulated individuals’ chronic regulation focus and situationally induced motivation to probe how the “fit” and “non-fit” of both motivational systems affected the decision makers’ visual information processing. Hypothesis 2 states that the chronic and situational regulatory focus interact on the information processing, and regulatory fit of chronic and situational focus can strengthen the individual’s motivation for their preferred information processing, whereas the non-fit can weaken the motivation.

## Experiment 1

### Materials and Methods

#### Subject Screening

Two hundred undergraduates (132 females and 68 males, mean age = 21.1 years) from Henan Normal University were tested by the Chinese version of Regulatory Focus Questionnaire (RFQ) (Yao et al., 2008). The RFQ consists of prevention subscale and promotion subscale. Both subscales exhibited good internal reliability (α = 0.73 for the Promotion subscale; α = 0.80 for the Prevention subscale) and test–retest reliability (0.79 and 0.81, respectively, for the Promotion and Prevention subscale) (Higgins et al., 2001). The Chinese version of the Regulatory Focus Questionnaire also had good internal reliability, α = 0.66 for the Promotion subscale and α = 0.61 for the Prevention subscale. Subjects were sorted by the *Z*-value of the difference between their scores on both subscales. The top and bottom 15% of the individuals were selected as chronic promoters and preventers, respectively. Twenty subjects were excluded because of their reluctance to participate in the eye-movement program or failure of eye-movement recording. Hence, 40 subjects (26 females and 14 males, mean age = 21.3) ultimately took part in the eye-movement recording program. All had normal or corrected-to-normal vision and were naive to the purpose of the experiment. The subjects took part in the test of Regulatory Focus Questionnaire, were paid an hour’s credit for their psychological courses, and the subjects who completed the eye-movement tracking experiment were paid 10 Yuan for their time.

#### Apparatus

EyeLink 1000 desk eye tracker (SR Research Ltd., Canada) was used to record participants’ oculomotor activities. Only the right eye was measured, although viewing was binocular. The eye tracker sampled eye position every 1 ms; and the refresh rate and resolution of the display screen were 150 Hz and 1,024^∗^768 pixels, respectively. The distance between participants’ eyes and the computer screen was about 65 cm. Each character on the screen was in Song font and subtended at approximately a 0.74° visual angle. A chin rest was used to minimize head movements.

#### Materials and Design

Previous researches on multi-attribute decision tasks employed two different information presentation methods. One approach was to display all attributes information on all alternatives in a matrix table, and the other was to present all attributes of a certain alternative; the purpose was to induce separately the attribute-based and alternative-based processing patterns. However, the visual information presented to participants in these two methods was not equal, and in the matrix presentation method, it could not be ruled out that some participants still processed information based on alternatives. Furthermore, it was found that the number of alternatives and attributes influenced the processing strategies (Schulte-Mecklenbeck et al., 2017; Perkovic et al., 2018).

**FIGURE 1:**
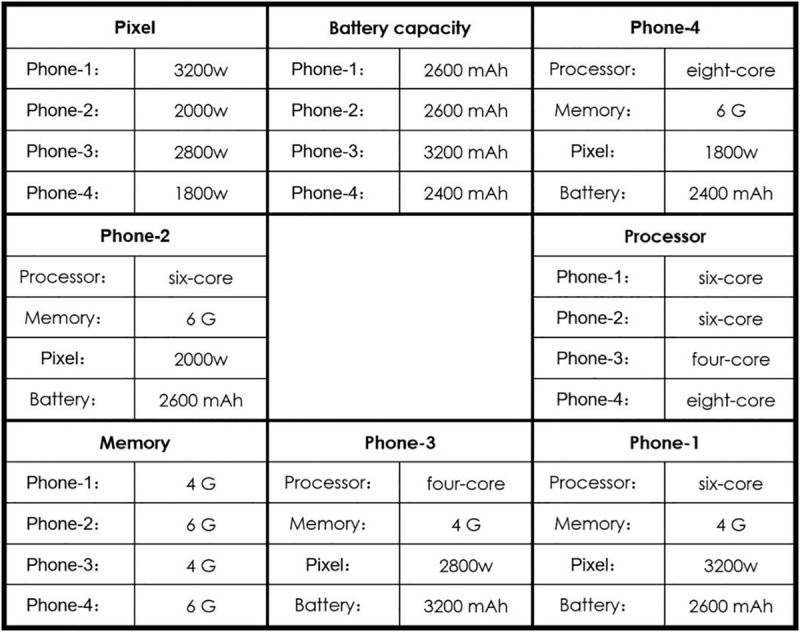
An example of a 3 × 3 grid information-processing task.

Bearing this in mind, in our study we set the number of alternatives and attributes for each trial to four and designed a 3 × 3 grid information-processing task (example in Figure 1), containing eight information cells (315 × 200 pixels each) and a fixation cell. The fixation cell was always placed in the center. The information cells were of two types: one type had the parameter values of four alternatives on a certain attribute, such as the pixel values of four mobile phones, matched with an attribute-based information processing pattern. The other type had four attribute values of an alternative, such as the pixel value battery capacity, memory size, and processor of mobile phone 1, matched with an alternative-based processing pattern. We arranged these information cells randomly on each trial to avoid the position effect. Each alternative in this study had an advantage attribute to ensure that the subjects could fully browse the information on all alternatives. Finally, we chose 15 products for the study, all of which were popular digital products or appliances with explicit attribute values.

#### Procedure

First, the subjects were screened. Two weeks later, the eye-movement experiment was performed on the screened subjects. The experimental procedure was as follows: (1) Nine-point calibration mode was completed to ensure tracking accuracy. (2) Prior to each trial, a drift check at the center of the screen was displayed to check calibration and then presented the central fixation cell to ensure that the center of the grid would be the starting gaze point. (3) Subjects were instructed to carefully watch each 3 × 3 grid and then select one product that they preferred to “purchase.” They could push any button on the keyboard to switch to the next display. (4) A selection screen appeared after the participants went over the grid by pressing any button on the keyboard. In the selection screen, the numbers 1–4, representing, respectively, four different alternatives, were displayed and the participants were instructed to press corresponding number keys on the keyboard to make selection. Based on results of our pretest, we set the maximum presentation duration of each 3 × 3 grid to 30 s. There were 15 trials, the first three of which were for practice and were excluded from the data analysis. We recorded each subject individually, and the total experiment time was around 20 min.

#### Analyses

The focus of our research was the features of cognitive processing of individuals with different chronic regulation focuses on the attribute- and alternative-based information. Consequently, we first divided the eight information cells into the corresponding eight areas of interest (AOIs) for each grid and then grouped them into two categories (attribute or alternative). Once the viewer’s fixation enters an AOI, the processing for this area begins. In almost all conditions, the viewer’s fixation could regress into this AOI again after processing the other AOI. So the processing prior to the first departure from the current AOI is referred as first-pass processing, which is related with the early stage of processing, and the afterward processing of this AOI is referred as second-pass processing, which is related with a later stage of processing. In the present study, we selected four eye-movement measures to reveal the features of different cognitive processing stages during the scene viewing: (1) first-fixation duration, referring to the duration of the initial fixation on a certain AOI during the first-pass processing; (2) first-pass time, or gaze duration, referring to the duration of viewing starting with the first fixation entering the current AOI until the fixation left this area for the first time; (3) frequency of regression-in, or the number of times the viewing shifted from the other AOI to the current AOI after the first-pass processing; (4) total viewing time ratio, or the ratio of total viewing time of the current AOI to the total viewing time of the entire matrix. In eye-movement studies (Rayner et al., 2008; Rayner, 2009), the first-fixation duration and the first-pass time are regarded as indicators of the earlier phase of viewing, and the combination of these two measures can reflect the degree of interest and early cognitive processing of the watchers. The frequency of regression-in is an indicator for the later phase of viewing, which mainly reflects the comparison and integration of viewed information in the later phase of processing. Total viewing time ratio is an overall processing indicator, reflecting the entire cognitive resources of the viewer’s input in the current AOI. A higher total viewing time ratio indicates that the viewer invested more cognitive resources and engaged in deeper processing of the current area.

### Results and Discussion

**FIGURE 2:**
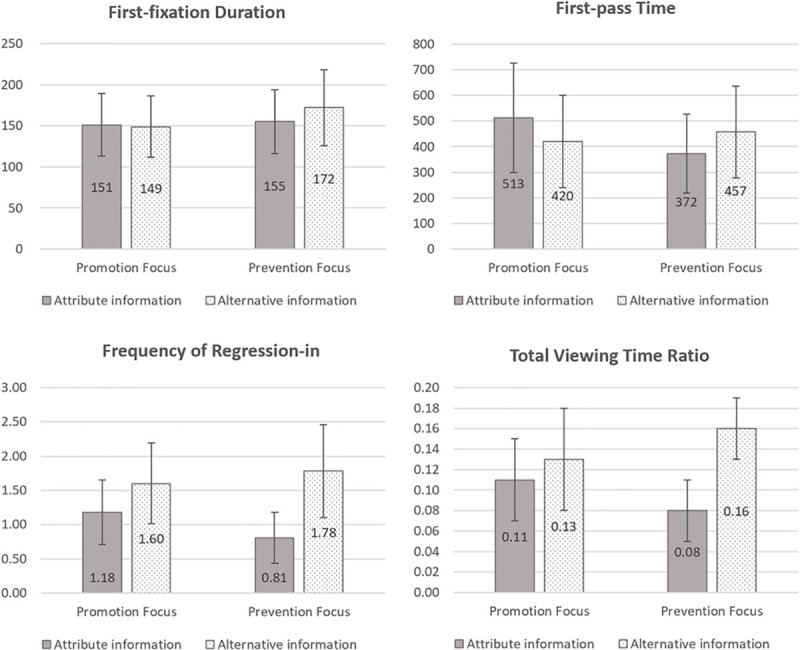
First-fixation duration, first-pass time, frequency of regression-in, and total viewing time ratio as a function of the chronic regulatory focus (promotion or prevention) and the type of information (attribute or alternative).

First, we compared the total viewing times of participants on each grid and found no significant difference between chronic promoters (*M* = 13,626.57, *SD* = 6,634.26) and chronic preventers (*M* = 12,332.85, *SD* = 5,233.33) where *F*_(1,38)_ = 2.55, *p* = 0.12. Then we examined the subjects’ eye-movement measurements in a 2 (chronic regulatory focus: promotion, prevention) × 2 (information type: based on attributes or alternatives) analysis of variance. Table 1 and Figure 2 show the descriptive statistics results of the four eye-movement measurements. The main effects of chronic regulatory focus in all measurements were not significant. The main effects of information type were significant for the first-fixation duration [*F*_(1,38)_ = 3.98, *p* = 0.05, η^2^ = 0.095], frequency of regression-in [*F*_(1,38)_ = 9.56, *p <* 0.01, η^2^ = 0.46], and total viewing time ratio [*F*_(1,38)_ = 16.21, *p <* 0.05, η^2^ = 0.30], which indicated that the two different information types set in this study were valid.

**TABLE 1:** Means and standard deviations of eye-movement measures in experiment 1 as functions of chronic regulatory focus and visual search pattern.

**Information type**	**Regulatory focus**	**FFD**	**FPT**	**FRI**	**TVR**
Attribute-based	Promotion	151 ± 38	513 ± 334	1.18 ± 0.47	0.11 ± 0.05
	Prevention	155 ± 39	372 ± 174	0.81 ± 0.37	0.08 ± 0.03
Alternative-based	Promotion	149 ± 39	420 ± 181	1.60 ± 0.79	0.13 ± 0.05
	Prevention	172 ± 46	457 ± 180	1.78 ± 0.88	0.16 ± 0.03

*FFD, first-fixation duration; FPT, first-pass time; FRI, frequency of regression-in; TVR, total viewing time ratio.*

The interactions between visual search patterns and chronic regulatory focus were significant for all four eye-movement measurements: first-fixation duration [*F*_(1,38)_ = 6.24, *p* = 0.01, η^2^ = 0.14], first-pass time [*F*_(1,38)_ = 6.85, *p* = 0.01, η^2^ = 0.15], frequency of regression-in [*F*_(1,38)_ = 5.34, *p <* 0.05, η^2^ = 0.12], and total viewing time ratio [*F*_(1,38)_ = 4.19, *p <* 0.05, η^2^ = 0.10]. These results showed that individuals with different chronic regulatory orientations manifested distinctive processing features during information coding before making a decision; furthermore, this difference was displayed from an early stage of information processing to the late integration stage.

The results of simple effect analysis further revealed that chronic preventers had longer first-fixation duration [*F*_(1,38)_ = 10.09, *p* < 0.05, η^2^ = 0.21], higher frequency of regression-in [*F*_(1,38)_ = 31.67, *p* < 0.001, η^2^ = 0.46], and a higher total viewing time ratio [*F*_(1,38)_ = 18.43, *p* = 0.01, η^2^ = 0.327] on the alternative-based cell than on the attribute-based cell. They also had slightly more first-pass time on the alternative-based cell [*F*_(1,38)_ = 3.13, *p* = 0.08, η^2^ = 0.08], although the difference did not reach a significant level. Obviously, chronic preventers steadily exhibit preference for alternative-based information. Conversely, the chronic promoters’ first-pass time on the attribute-based cell was slightly higher than on the alternative-based cell [*F*_(1,38)_ = 3.74, *p* = 0.06, η^2^ = 0.09], but the frequency of regression-in on the attribute-based cell was significantly lower than on the alternative-based cell [*F*_(1,38)_ = 5.56, *p* < 0.05, η^2^ = 0.13]. This interesting result indicates that the information processing of promoters may be more flexible. Specifically, the chronic promoters preferred attribute-based information in the early processing stage, but they obtained more types of information for comparison in the later coding stage. For this reason, there was no difference in the total viewing time ratio of chronic promoters between the two types of information, *p* > 0.10.

All the above results of multiple eye-movement measures reveal that the chronic self-regulatory system has a significant impact on the individuals’ visual information processing. Chronic preventers consistently prefer an alternative-based information processing pattern, whereas chronic promoters do not exhibit a clear preference between the two types of information, and their information processing is more flexible and comprehensive.

## Experiment 2

To explore the interaction effects of chronic and situational regulatory focus on information processing for decision making, we manipulated the types of subjects’ chronic and situational regulatory focuses simultaneously in this experiment.

### Materials and Methods

#### Subject Screening

We administered the Chinese version of Regulatory Focus Questionnaire (Yao et al., 2008) to 600 college students from Henan Normal University. Ultimately, we screened 80 subjects (32 males and 48 females, mean age = 21.8), including 40 chronic promoters and 40 chronic preventers, using a similar screening method as in Experiment 1. The standards of remuneration for participants in experiment 2 were similar with that in experiment 1, except that the subjects who participated in the eye-movement tracking experiment received 15 Yuan as reward.

#### Procedure and Materials

We randomly divided each chronic-focus group of subjects into two groups to be tested with induced situational promotion or prevention focus, respectively, by which we obtained four groups of subjects (20 subjects in each group): chronic promoters in a promotion situation (CPro-SPro), chronic promoters in a prevention situation (CPro-SPre), chronic preventers in a prevention situation (CPre-SPre), and chronic preventers in a promotion situation (CPre-SPro).

There were three conditions to induce the desired situational focus: recall-report task, maze task and the verbal motivation regarding remuneration. In the recall-report task, we instructed the subjects to recall and write down some ideal things such as ideal job or ideal mate, to induce situational promotion motivation, or some things that should be done such as the responsibility or obligation, to induce situational prevention motivation (Chernev, 2004; Hamstra et al., 2014). In the pencil-and-paper maze task, the subjects were required to use the pen to draw a route out of the maze under different instructions. One guideline induced a promotion focus by requiring subjects to guide a mouse in the middle of the maze to escape so that it could eat the cheese at the labyrinth exit. The other guideline induced a prevention focus by directing the subjects to lead the mouse to escape so it could avoid owls that hovered over the maze waiting to catch prey (Friedman and Förster, 2001). The last situational condition was verbal motivation, in which the induced promoters were informed, “You have a fixed amount of remuneration, and you will get additional rewards for excellent performance”; and the induced preventers were told, “You have a fixed amount of remuneration, but poor performance will reduce some of your remuneration” (Hamstra et al., 2014; Sassenberg et al., 2014).

The eye-movement experiment was performed on each subject after the three situational conditions. The experimental procedures, materials, and data analyses were the same as in experiment 1, and the total time taken for experiment 2 was about 1.5 h.

### Results and Discussion

A 2 (chronic regulatory focus) × 2 (information type) × 2 (situationally induced regulatory focus) analysis of variance was conducted on the subjects’ eye-movement measurements. Table 2 shows the results of the descriptive statistics. There were no significant main effects of chronic regulatory focus except for the measure of frequency of regression-in [*F*_(1,76)_ = 7.41, *p* < 0.05, η^2^ = 0.09], indicating that the type of chronic regulatory focus alone has almost no significant impact on information coding. Our manipulation of the visual information type in this experiment was also effective, as shown by the significant main effect of information type on first-fixation duration [*F*_(1,76)_ = 4.59, *p* = 0.035, η^2^ = 0.06] and frequency of regression-in [*F*_(1,76)_ = 24.32, *p* = 0.001, η^2^ = 0.24]; total viewing time ratio was marginally significant [*F*_(1,76)_ = 3.47, *p* = 0.06, η^2^ = 0.05].

**TABLE 2:** Means and standard deviations of eye-movement measures in experiment 2 as functions of chronic and situationally induced regulatory focus and visual search pattern.

**Information type**	**Regulatory focus**	**FFD**	**FPT**	**FRI**	**TVR**
Attribute-based	CPro-SPro	176 ± 33	576 ± 284	1.41 ± 0.53	0.14 ± 0.04
	CPro-SPre	154 ± 41	467 ± 187	0.92 ± 0.43	0.10 ± 0.03
	CPre-SPre	165 ± 32	510 ± 176	1.31 ± 0.44	0.12 ± 0.03
	CPre-SPro	181 ± 28	448 ± 154	1.21 ± 0.39	0.10 ± 0.04
Alternative-based	CPro-SPro	183 ± 44	531 ± 178	1.40 ± 0.59	0.11 ± 0.04
	CPro-SPre	161 ± 42	443 ± 166	1.35 ± 0.75	0.13 ± 0.03
	CPre-SPre	163 ± 35	483 ± 143	1.63 ± 0.76	0.12 ± 0.03
	CPre-SPro	193 ± 24	494 ± 123	2.16 ± 0.97	0.15 ± 0.04

*CPro-SPro, chronic promoters under situation promotion; CPro-SPre, chronic promoters under situation prevention; CPre-SPre, chronic preventers under situation prevention; CPre-SPro, chronic preventers under situation promotion.*

Consistent with the results of experiment 1, the interactive effects of chronic self-regulatory focus and visual information type were significant for frequency of regression-in [*F*_(1,76)_ = 6.07, *p* = 0.016, η^2^ = 0.07] and marginally significant for total viewing time ratio [*F*_(1,76)_ = 3.18, *p* = 0.08, η^2^ = 0.04]. Chronic-preventers had a greater frequency of regression-in [*M* = 1.90, *SD* = 0.12; *F*_(1,76)_ = 27.34, *p* = 0.001, η^2^ = 0.26] and a higher total viewing time ratio [*M* = 0.13, *SD* = 0.01; *F*_(1,76)_ = 6.65, *p* = 0.01, η^2^ = 0.08] for the alternative-based cell than for the attribute-based cell (*M* = 1.26, *SD* = 0.41 and *M* = 0.11, *SD* = 0.01, respectively). These results suggest that chronic preventers distinctly prefer alternative-based information.

The interaction of situationally induced orientation and visual information type was significant for frequency of regression-in [*F*_(1,76)_ = 9.82, *p* = 0.002, η^2^ = 0.11] and total viewing time ratio [*F*_(1,76)_ = 14.86, *p* = 0.001, η^2^ = 0.164]. In the induced prevention situation, frequency of regression-in [*M* = 1.75, *SD* = 0.12; *F*_(1,76)_ = 32.52, *p* = 0.001, η^2^ = 0.30] and total viewing time ratio [*M* = 0.14, *SD* = 0.33; *F*_(1,76)_ = 16.34, *p* = 0.001, η^2^ = 0.177] for the alternative-based cell were significantly higher than for the attribute-based cell (*M* = 1.06, *SD* = 0.07 and *M* = 0.10, *SD* = 0.34, respectively). The results revealed that temporary regulatory focus stimulated by experimental tasks affects information processing. Furthermore, this effect is basically consistent with the chronic focus: preventers favored alternative-based information, and promoters expressed almost no difference in the processing of the two types of information. Similarly, the results of eye-movement measurements indicate that the impact of situationally induced motivation on information processing is mainly reflected in the later stage of processing.

We also found significant interaction of chronic orientations and situationally induced regulatory focus in the first-fixation duration, frequency of regression-in, and total viewing time ratio [*F*_(1,76)_ = 8.70, *p* = 0.004, η^2^ = 0.103; *F*_(1,76)_ = 4.45, *p* = 0.04, η^2^ = 0.06; and *F*_(1,76)_ = 5.36, *p* = 0.02, η^2^ = 0.07, respectively]. These results reveal that the situational regulation focus modulates the information processing of individuals, whether they are chronic promoters or preventers. The simple effect analysis showed that the chronic promoters had significantly longer first-fixation duration and higher frequency of regression-in and total viewing time ratio under induced promotion situation than that under prevention situation [*M* = 179 vs. *M* = 158, *F*_(1,76)_ = 4.13, *p* = 0.05, η^2^ = 0.05, and *M* = 1.41 vs. *M* = 1.14, *F*_(1,76)_ = 4.94, *p* = 0.03, η^2^ = 0.09, and *M* = 0.13 vs. *M* = 0.11, *F*_(1,76)_ = 3.98, *p* = 0.05, η^2^ = 0.08, respectively], Whereas, the chronic preventers had significantly longer first-fixation duration and higher frequency of regression-in under induced prevention situation than those under promotion situation [*M* = 187 vs. *M* = 164, *F*_(1,76)_ = 4.57, *p* = 0.04, η^2^ = 0.06, and *M* = 1.69 vs. *M* = 1.47, *F*_(1,76)_ = 4.54, *p* = 0.03, η^2^ = 0.08, respectively].

**FIGURE 3:**
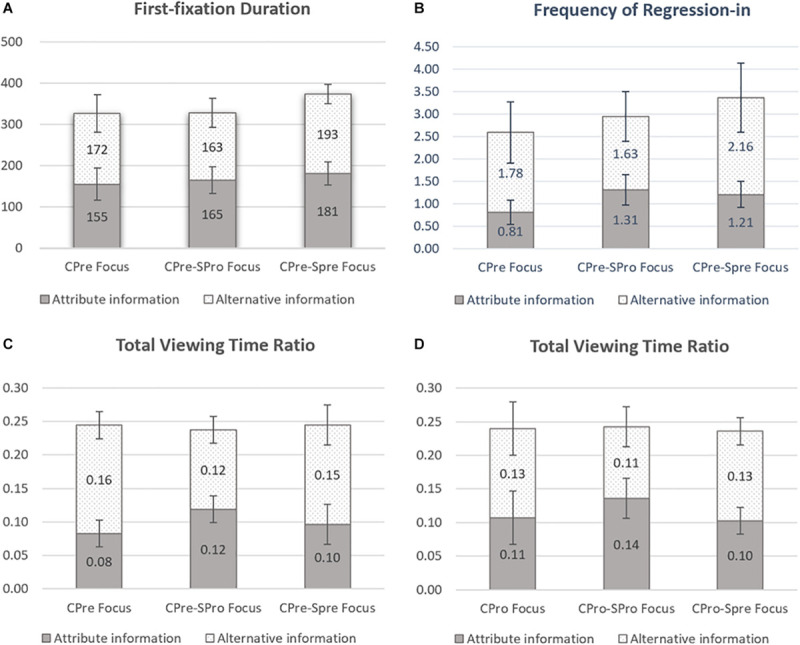
First-fixation duration **(A)**, frequency of regression-in **(B)**, and total viewing time ratio **(C)**, as a function of the prevention focus and the type of information; and total viewing time ratio **(D)**, as a function of the promotion focus and the type of information.

To further analyze the influence of the compatibility or fit of chronic and situational regulatory focus on individual visual information processing, we integrated the data of experiment 1 and experiment 2 and separately compared the differences in information processing between chronic promoters (CPro), chronic promoters in a promotion situation (CPro-SPro), and chronic promoters in a prevention situation (CPro-SPre), as well as between chronic preventers (CPre), chronic preventers in a prevention situation (CPre-SPre), and chronic preventers in a promotion situation (CPre-SPro). We performed a 2 (information-type) × 3 (regulatory focus) analysis of variance on four eye-movement measurements under the promotion-focus condition and the prevention-focus condition, respectively. Figure 3 shows the significant results.

We found that under the prevention-focus condition, the interaction of information type and regulatory focus is significant in first-fixation duration [*F*_(2,57)_ = 3.38, *p* = 0.04, η^2^ = 0.11; see Figure 3A], frequency of regression-in [*F*_(2,57)_ = 4.85, *p* = 0.01, η^2^ = 0.15; see Figure 3B], and total viewing time ratio [*F*_(2,57)_ = 8.21, *p* = 0.001, η^2^ = 0.22; see Figure 3C]. Under the promotion-focus condition, the interaction of information type and regulatory focus is significant in total viewing time ratio [*F*_(2,57)_ = 3.55, *p* = 0.04, η^2^ = 0.11; see Figure 3D].

Similar to chronic preventers, CPre-SPre subjects show more frequency of regression-in and total viewing time ratio for the alternative-based cell than for the attribute-based cell (*p* < 0.05 for all). However, there are no differences between the two cells in all measures for the CPre-SPro subjects. Furthermore, the CPre-SPre subjects show more first-fixation duration and frequency of regression-in with the alternative-based cell than do chronic preventers and CPre-SPro subjects (*p* < 0.05 for all). Additionally, with the attribute-based cell, CPre-SPre subjects show more first-fixation duration and frequency of regression-in than do chronic preventers (*p* < 0.05 for all). These results indicate that CPre-SPre subjects not only prefer to process the alternative-based information but also exhibit deeper processing of both types of information compared with chronic preventers.

On the other side, CPro-SPro subjects show a greater total viewing time ratio with the attribute-based cell than with the alternative-based cell (*p* < 0.05). Contrarily, CPro-SPre subjects show a greater total viewing time ratio for alternative-based cell than for attribute-based cell (*p* < 0.05). Furthermore, CPro-SPro subjects show a greater total viewing time ratio for attribute-based cell than do CPro-SPre subjects (*p* < 0.05). Comparing CPro-SPro subjects with chronic promoters, we found similar but not significant trends.

Integrating the above results, it can be inferred that information processing is deeper when the chronic regulation focus matches the situationally induced focus. However, when the focuses do not match, the extent of information preference is lowered, which probably means that viewers give similar attention and effort to both information areas.

## General Discussion

### The Regulatory Focus and Information Processing Features

The results of multiple eye-movement measurements in this study consistently reveal that both the chronic and the situationally induced regulatory focuses separately affect individuals’ visual information processing. Individuals with differing chronic regulation focuses show distinctively different characteristics in information processing. Chronic preventers exhibit their preference for alternative-based information at the initial stage of processing, which we discovered in the measurement of first-fixation duration. Furthermore, this trend manifests steadily in the later stages of processing, which we see from the indexes of the frequency of regression-in and total viewing time ratio. This more consistent and stable information-preference strategy aids viewers to obtain more accurate and specific information and to avoid mistakes, which fits the primary concern of an individual with prevention-focused vigilance. Promotion-focused individuals, on the other hand, seem to use a compensation strategy in which they slightly prefer attribute-based information in the early processing stage, but they deeply process alternative-based information in the later stage. These findings indicate that promotion-focused individuals have a more flexible and comprehensive information processing pattern in multi-attribute decision-making tasks, ensuring that they can process information at a more global level, supporting hypothesis 1. The results are consistent with the findings of previous studies (Chernev, 2004; Lee et al., 2009; Florack et al., 2010). This is because a more comprehensive information search mode should facilitate opportunities for recognition and reduce omissions, matching the characteristics of an individual with promotion-focused eagerness. These results also support the suggestion of Derryberry and Tucker (1994) and Derryberry and Reed (1998) that avoidance-related motivational states narrow the scope of perceptual attention, yielding visual focus on local details, whereas approach-related motivational states exhibit the opposite.

Situationally induced motivational orientation has a roughly similar effect on information processing. This finding is concordant with the results of Peng et al. (2019), where chronic regulation orientation and situational orientation have the same effect on self-framing. This was evidenced by the promotion-focus subjects, compared with prevention-focus subjects, being more willing to employ positive words to express ambiguous decision-making information and producing a more positive self-framework. The self-framework effect explores how decision makers process and code information before making decisions, which also reflects the features of the initial stage of decision making, i.e., coding. The results of converging studies have revealed that the regulatory focus system has a significant effect on the information coding stage of decision making. According to Higgins (2000), individuals with distinctive motivation orientations adopt their own preferred behavioral strategies in pursuit of goals, thus achieving the effect of regulatory fit. Furthermore, the match of the strategy and the goal allows individuals to experience a positive and appropriate feeling about their current behaviors, which further strengthens their behavioral motivation, enhances job performance, and magnifies this emotional experience (Cesario et al., 2004; Aaker and Lee, 2006). Likewise, in the present study, we observed strong motivation and engagement with preferred information through the individuals’ oculomotor activities, although we did not directly assess their feelings about behavior and decision making.

### The Fit and Non-fit of Chronic and Situational Regulatory Focus

The results reveal that experimental regulatory situations can modulate the information processing of individuals with differing chronic regulation focuses. Furthermore, for the first time, chronic focus was used as the benchmark condition to compare with matched and mismatched conditions. We found that individuals tend to put more effort toward processing their preferred information when their chronic focus matches the situationally induced focus, consistent with hypothesis 2. These findings also strongly support the regulatory fit hypothesis from another perspective, i.e., the match or fit of chronic and situational focus. Similar results come from the study of Keller and Bless (2006), which revealed that chronic and situational focus have a significant interactional effect on cognitive performance; specifically, individuals’ test performance improves when the situational focus matches the chronic focus. Thus, the fit between chronic and situational focus impacts not only information processing, but also cognitive performance.

The comparison of the three conditions (chronic focus alone, chronic-situational match, and chronic-situational mismatch) allows us to discover the information-processing features under regulatory non-fit. Our results demonstrate that individuals, especially chronic preventers, are inclined to pay similar attention and effort to both information areas when they experience focus non-fit, which means that they process information on a more global level to achieve more overall information. It seems that individuals experiencing non-fit need to reallocate cognitive resources to address these discrepancies (Keller and Bless, 2006). According to research (Vaughn et al., 2006; Fridman et al., 2016) on the effect of regulatory non-fit on medical judgment, non-fit makes individuals less confident in their initial judgment and prods them to consider more thoroughly the advice presented by physicians. Similar to medical judgment tasks, the information processing behaviors and judgments in the multi-attribute decision tasks also reveal that regulatory non-fit makes individuals less confident and more uncertain in their initial judgments based on the preferred information, which in turn motivates them to pay more attention to another type of information, further supporting hypothesis 2.

## Conclusion

In conclusion, we demonstrated that, in the multi-attribute decision tasks, the regulatory focus, regardless of chronic or situational focus, significantly influences the information-processing patterns. Individuals with prevention-focused vigilance constantly prefer alternative-based information, whereas, promotion-focused individuals have a more flexible and comprehensive processing patterns. The regulatory fit of chronic and situational focus tends to intensify the individuals’ cognitive-processing motivation and depth for their preferred information. Meanwhile, regulatory non-fit results in non-preferred processing for alternative and attribute information by de-intensifying the confidence on the initial judgment from preferred information.

Our study has some limitations but also provides fruitful suggestions for future research. First, our subjects were screened from a large pool, and all had a significant chronic regulatory focus. This selection enhances the experimental effects but could miss some valuable discoveries coming from the middle range of individuals. Future research should consider individuals’ chronic regulatory focus as a continuous variable. Second, the study revealed information-processing features only via eye-movement measurements. Although eye movement has been proved to be a valid measure of the spatial distribution of attention, future research would be more valid and valuable by integrating the results of eye-movement recording and the participants’ verbal reports about their processing of different types of information. Third, the presentation of each 3 × 3 grid was restricted to 30 s; this potentially influenced the participants’ information processing, although our pretest results showed that 30 s was long enough to process the 3 × 3 grid.

## Data Availability Statement

The datasets generated for this study are available on request to the corresponding author.

## Ethics Statement

The studies involving human participants were reviewed and approved by the Henan Normal University. The patients/participants provided their written informed consent to participate in this study.

## Author Contributions

JX supervised the collection of the data and wrote this manuscript. XJ and WL collected and analyzed the data. All authors contributed to the article and approved the submitted version.

## Conflict of Interest

The authors declare that the research was conducted in the absence of any commercial or financial relationships that could be construed as a potential conflict of interest.
